# Effect of a Low-Moderate Exercise Program on Dysmetabolism in Older Adults: Results of a Randomized Controlled Trial

**DOI:** 10.3390/nu14163337

**Published:** 2022-08-15

**Authors:** Fernanda Velluzzi, Giulia Cossu, Michele Fosci, Roberta Montisci, Rosanna Zaccheddu, Luigi Minerba, Mario Musu, Elisa Pintus, Dario Fortin, Ferdinando Romano, Cesar Ivan Aviles Gonzalez, Paola Melis, Andrea Deledda, Andrea Loviselli, Mauro Giovanni Carta

**Affiliations:** 1 Department of Medical Sciences and Public Health, University of Cagliari, 09124 Cagliari, Italy; 2 Department of Psychology and Cognitive Science, University of Trento, 38123 Trento, Italy; 3 Department of Public Health and Infectious Diseases, Unitelma Sapienza University, 00185 Rome, Italy; 4 Departamento de Facultad de Ciencias de la Salud, Universidad Popular del Cesar, Valledupar 200002, Colombia

**Keywords:** physical exercise, older adults, dysmetabolism, overweight, nutrition, healthy diet, moderate exercise, cardiovascular risk

## Abstract

Physical exercise has been shown to improve dysmetabolism in older adults, reducing cardiovascular risk, while its role in preventing dysmetabolism is less known. Moreover, most of the trials use exercise programs that are difficult to put into daily practice. The purpose of this Randomized Controlled Trial (RCT) was to evaluate the effectiveness of a 3-month moderate exercise program in improving or preventing dysmetabolism in 120 older adults, randomly selected for the exercise program (experimental group) or cultural activities (control group). None of the subjects were following a hypocaloric diet, and all of them reported healthy eating habits. Anthropometric (Body Mass Index (BMI) and Waist Circumference (WC)) and metabolic variables (fasting plasma glucose (FPG), High-Density Lipoprotein Cholesterol (HDL-C), and triglycerides (TG)) were assessed at baseline (T0) and at the end of the trial (T1). Dysmetabolism was defined by the presence of an increased WC plus at least two metabolic alterations. At T0, the two groups did not differ by sex, age, education, BMI, WC, FPG, HDL-C levels, and prevalence of dysmetabolism. The mean BMI value indicated overweight, and WC values were higher than the cut-off. At T1, a slight reduction in the number of people with dysmetabolism was found only in the experimental group. However, none of the individuals without dysmetabolism at T0 in the experimental group developed it at T1, while 11.4% developed it in the control group (*p* = 0.032). This study highlights that a moderate exercise program, accessible in daily practice, can prevent dysmetabolism in older adults, even while being overweight, while if dysmetabolism is already present, more prolonged combined nutritional and exercise interventions will be needed.

## 1. Introduction

Physical activity, combined with healthy dietary patterns in lifestyle intervention, plays a key role both in the prevention of Type 2 diabetes mellitus (T2DM) in people at high risk [1,2] and in its management [3,4]. In particular, structured exercise consisting of aerobic, resistance training, or their combination is associated with HbA1c reduction in patients with T2DM [5]. Moreover, acute or chronic exercise, both continuous or accumulated, has been found to reduce postprandial glucose (PPG) or insulin levels in diabetic and non-diabetic adults, and this effect may be improved by considering exercise-nutrient interactions [6]. However, accumulated moderate exercise showed a greater effect on same-day PPG in non-diabetic individuals, while there was no difference between continuous or accumulated training on same-day post-prandial insulin or triglycerides levels [7]. In addition, it has been established that moderate intensity continuous training ameliorates dysmetabolism in patients with metabolic syndrome (MS), reducing the cardiovascular risk (CVR) [8]. In fact, regular physical exercise has been shown to reduce visceral adiposity, the central component of MS which promotes a set of metabolic alterations and a chronic inflammatory status, increasing CVR [9]. Furthermore, it has been hypothesized that physical activity may have beneficial effects, similar to drug interventions, on mortality and the secondary prevention of some cardiovascular diseases (CVDs) and diabetes [10]. On the other hand, a sedentary lifestyle may have an independent effect on CVR, as indicated by clinical [11] evidence. Due to its favorable effects on body composition, metabolic control, and inflammation, even if started in old age, physical exercise should be recommended as an effective and safe strategy in the management of cardiometabolic diseases, also in the elderly population [12]. Aging is a complex process characterized by a decrease in functional performance and an increased morbidity, both associated with a worsening quality of life. Among chronic diseases, T2DM is one of the most prevalent in older people [13], and this condition shows a close relationship with frailty and [14] sarcopenia. Insulin resistance, the main pathophysiological feature of T2DM, commonly preceding the clinical onset of the disease, has been associated with low lean muscle mass and frailty in elderly population [15]. MS has also been associated with frailty [16], although not with unique data [17]. Besides the aforementioned cardiometabolic benefits [12], physical activity, along with adequate nutrition [18], is a particularly important component of lifestyle interventions in older people, also to its beneficial effects in preventing or relieving several age-related conditions such as pain, sarcopenia, osteoporosis, cognitive impairment, and in promoting healthy aging. More specifically, exercise programs for older adults should include aerobic, strength, balance, and flexibility components [19].

As for aerobic exercise, one of the main benefits is the improvement in oxidative capacity and in cardiorespiratory fitness, resulting in an increased fatigue resistance. Different activities have proven to be effective in achieving these goals, among which walking is the easiest and most feasible [20]. Nonetheless, exercise programs that combine aerobic and resistance training may confer the health benefits of both types of exercise. Indeed, resistance training provides several health benefits in older adults, improving in particular body composition [21,22,23], chronic inflammation, and glucose metabolism [24,25]. The beneficial effects of resistance training on glucose or lipid alterations play an important role in the management of chronic cardiovascular or metabolic diseases associated with aging [26]. Moreover, the improvement of body composition, and especially the increase of lean mass, the better flexibility and balance, after a resistance training program, have been associated with a significantly improved quality of life in older adults [27]. Finally, it has been reported that concurrent training results in a significant improvement of WC, blood pressure or HDL-Cholesterol in older patients affected by MS [28]. Thus, the ideal exercise program in older adults should include aerobic, resistance, as well as flexibility, and balance training, with such intensity, volume, and duration as to obtain the maximum benefit [29].

Based on these principles, specific guidelines have been recently developed for older adults [30], although exercise should be individually tailored to the characteristic of each person [29]. However, there are not sufficient data on the effects of different combined protocols on MS outcomes in older adults [31]. It has been reported that a 12-week intervention including resistance and aerobic training conferred cardiometabolic benefits with both high and moderate intensity aerobic training in older adults with MS, but the authors concluded that the results needed to be confirmed with long-term programs [32].

Moreover, despite the well-documented beneficial effects of physical exercise on metabolic diseases, the role of moderate exercise in preventing dysmetabolism is less evident. There are limited or insufficient data on how much lifestyle changes, diet, and more specifically exercise could impact dysmetabolism, or lower the risk of cardiovascular and microvascular complications in patients with type 2 diabetes mellitus [33,34].

Furthermore, there is a limitation in translating experimental data into daily life guidelines, as most of the trials conducted on exercise in the elderly, a vulnerable age range for metabolic diseases, use exercise of high intensity and with an excessive number of weekly sessions [35,36]. On the one hand, this makes the results of exercise training poorly extensible into daily practice because physical exercise of this intensity cannot be conducted without very close specialist control, and on the other hand, it excludes elderly people with mild chronic disorders [37]. This represents a relevant issue when we consider that adherence to physical exercise in the elderly has been reported to be usually low, whereas, given the several health benefits it carries, much more effort should be targeted at increasing it [20].

The purpose of this study is to verify if a moderate exercise program can improve or prevent the early indices of dysmetabolism in older adults. The exercise was conducted with a modality (three sessions a week away from intense activity) that allows the extension of that model to daily practice in elderly people living in the community and who may also suffer from chronic disorders of mild to moderate level.

## 2. Materials and Methods

### 2.1. Study Design

This study is a 12-week Randomized Controlled Trial (RCT) registered at ClinicalTrials.gov, NCT03858114, aimed at evaluating the effects of an intervention based on moderate physical exercise on different aspects of biopsychosocial health in a sample of older adults living in the community [35]. The results concerning some outcomes of the trial have already been published [37,38].

### 2.2. Participants

One hundred and twenty older adults, recruited through public notices, were randomly drawn to a 12-week program of three sessions per week of moderate-intensity, mixed aerobic-anaerobic, physical exercise (experimental group) or a control protocol consisting of leisure/cultural activities, without the involvement of physical activity (control group).

### 2.3. Inclusion Criteria

Older adults (65 years and older) of both genders, living at home and considered eligible to attend non-competitive physical activity through a medical sport examination performed at the sports medicine center of the University of Cagliari.

### 2.4. Exclusion Criteria

Unsuitability for moderate exercise due to medical illness, severe obesity, lifetime diagnosis of severe psychiatric disturbances or organic brain disease, and involvement in recent months in a program of physical exercise.

### 2.5. Pre-Treatment Assessment

All participants underwent a 2-week pre-treatment assessment, concerning their physical, medical, and psychological status, and received detailed information about the study. Then, after signing the informed consent, they were randomly assigned to the exercise or the cultural protocol.

The pre-treatment medical assessment showed that none of the participants in the study were following a hypocaloric diet, and all of them reported healthy eating habits. In particular, a brief nutritional history regarding the food intake, highlighted that all participants had a balanced diet characterized by a regular daily consumption of vegetables, fruit, extra-virgin oil, and a low consumption of meat, high-fat dairy, or processed foods according to the definition of a healthy diet [39,40]. These habits reflect a dietary pattern in line with the Mediterranean model, as observed among adult people living in the Mediterranean area [41,42,43]. In addition, 1 of the 10 items of the Brief Social Rhythms Scale (BSRS), a measure of the regulation of biological rhythms included in the RCT, concerns the regularity of meals; the results, previously published, showed low scores, suggestive of regularity in daily activities including eating [44].

### 2.6. Exercise Protocol

A detailed description of the exercise has already been published [35]. Briefly, physical activity was administered in 3 sessions per week at 60–80% of the Heart Rate Reserve, with a mix of aerobic and anaerobic training and strength and balance exercises. Each session included an initial phase of warm-up (10 min), an active central phase (45 min), and a final cool down phase (10 min). Heart Rate, registered at baseline as a 3-day mean, was monitored continuously during activity.

### 2.7. Anthropometric and Metabolic Assessment

The anthropometric and metabolic assessment was performed at baseline (T0) and at the end of the trial (T1).

As for anthropometric evaluation, performed by an expert nutritionist, body weight (kg) was measured with an impedance scale (Model TANITA BC420 MA, Amsterdam, The Netherlands), height (cm), was measured by a stadiometer (SECA, Hamburg, Germany), and the BMI was calculated as body weight divided by height squared (kg/m^2^). Waist circumference (WC) was measured with a non-stretchable tape measure to the nearest 0.1 cm [45]. All measurements were taken with subjects barefoot and wearing light clothes.

A peripheral venous blood sample was drawn after overnight fasting (≥10 h), and the metabolic analysis was performed with standard methods in the same laboratory. For all the metabolic variables, including fasting plasma glucose (FPG), triglycerides (TG), and High-Density Lipoprotein Cholesterol (HDL-C), the International Diabetes Federation (IDF) cut-off was considered [46].

Dysmetabolism was defined by the presence of a WC value higher than the sex-specific cut-off (≥ 94cm in males, ≥ 80 cm in females), plus at least 2 of the following criteria:

(2a) fasting plasma glucose (FPG) ≥ 100 mg/dL

(2b) triglycerides (TG) ≥ 150 mg/dL

(2b) High-Density Lipoprotein Cholesterol (HDL-C) < 40 mg/dL males, < 50 mg/dL females.

In addition, the Blood Pressure (BP), Systolic (SBP), and Diastolic (DBP) measurement (normal values < 130/85 mmHg), included in the pre-trial medical sport evaluation, was performed.

### 2.8. Statistical Analysis

The comparison of the variable measured by nominal data (sex) between groups at T0 was performed using a chi square test. The comparison of variables measured by numerical data (age, education, BMI, WC, metabolic biochemical markers, BP), and expressed as mean value ± standard deviation (M ± SD), between groups at T0 was performed using a one-way ANOVA. The comparison of people with a dysmetabolism before and after the trial in the two groups was carried out by non-parametric approach considering time (T0, T1) and group according to the Siegel and Castellan method [47]. The risk of dysmetabolism at T1 among people who did not have it at T0, was calculated by means of a chi square test. For all tests performed on SPSS software (v. 28.0.1.0., IBM, Armonk, NY, USA), a *p* value (*p*) < 0.05 was considered statistically significant.

## 3. Results

The active intervention (12 weeks exercise or cultural protocol) was completed by, 52 people (87%) of the experimental group, and by 53 people (88%) of the control group.

Table 1 shows that the two groups did not differ at T0 by sex, age, years of education, BMI, WC, or by FPG and HDL-C levels. Higher TG levels were found in the experimental group than in the control group (103.03 ± 40.68 versus 86.12 ± 28.61, F = 6.089, df 1;103, *p* < 0.015). Moreover, mean SBP and DBP values were not significantly different between the two groups. In particular, SBP mean values were 134.20 ± 16.91 mmHg in the experimental group, and 129.77 ± 11.80 mmHg in the control group (*p* = 0.15); DBP mean values were 80.65 ± 8.07 mmHg in the experimental group, and 77.56 ± 7.74 mmHg in the control group (*p* = 0.07).

Regarding the BMI, both groups presented a mean value comprised in the range of overweight values with a standard deviation value reaching the obesity boundary, while WC mean value was higher than the sex-specific cut-off in males and females of both groups.

Table 2 shows the results of the effectiveness of exercise in the improvement of dysmetabolism already present at baseline in both groups (A) or prevention of dysmetabolism if it was not present at baseline (B).

In particular, Table 2 indicates that at baseline, dysmetabolism (increased WC values plus at least two metabolic alterations) was found in 28.3% and in 16.9% of people of the experimental group and control group, respectively; this difference was not statistically significant. In addition, all people affected by dysmetabolism, and over 60% of people without dysmetabolism also had mild hypertension controlled with medical treatment.

Table 2 also indicates that at the end of the trial (T1) a reduction in the number of people with dysmetabolism was found only in the experimental group after the exercise program, although it was not significant. No changes were observed before and after the trial in the control group (χ^2^ = 1.907, *p* = 0.167, analysis of variance for nominal data considering time and groups). Regarding people who did not show dysmetabolism at baseline (T0), none of the individuals of the experimental group developed it at T1 (0/37). Instead, 5 out of 44 (11.4%) people in the control group without dysmetabolism at baseline developed it at T1 (χ^2^ = 4.599, *p* = 0.032).

Moreover, the anthropometric assessment at T1, did not show any significant change in the mean values of WC, considered the main criterion of dysmetabolism, compared to those observed at baseline, in both the experimental and control group. This result was obtained in the whole sample (92.7 + 11.5 cm in the experimental group, and 92.3 + 11.7 cm in the control group; *p* = 0.87), and in both sexes (Males: 96.8 + 8.6 cm in the experimental group, and 98.8 + 8.6 cm in the control group; *p* = 0.5; Females: 89.2 + 12.5 cm in the experimental group, and 88.9 + 12.8 cm in the control group; *p* = 0.9).

## 4. Discussion

The first result of this study shows that a 12-week moderate-level, combined exercise program, conducted in three sessions per week, was not able to significantly improve dysmetabolism in a sample of older adults living in the community, in comparison with a control group assigned to a cultural program, which did not include any physical activity. Indeed, at the end of the trial, despite the reduction observed in the experimental group, the number of people who had shown dysmetabolism at baseline, remained statistically unchanged in both groups. The two study groups were statistically balanced for all the variables considered, except for the level of triglycerides which was slightly higher in the experimental group. Nevertheless, this randomly generated difference goes against the hypothesis of the study (i.e., a higher level of triglycerides could favor the onset of dysmetabolism), and therefore does not affect the validity of the results.

More specifically, we defined dysmetabolism as the association of an elevated WC value with at least two alterations among hyperglycemia, hypertriglyceridemia, or low HDL-C values, which, along with hypertension, represent the diagnostic criteria for metabolic syndrome according to IDF [48].

Metabolic syndrome is a condition with a globally growing prevalence [49], associated with an increased risk of cardiovascular disease and all-cause mortality [50]. Its development has shown an inverse correlation with cardiorespiratory fitness [51], and low physical activity level [52].

Several studies evaluated the effectiveness of physical exercise programs on MS, but the complexity of this condition, which involves a cluster of risk factors, makes for a difficult analysis of the clinical relevance of the results, which are often significant only on individual factors of MS [53].

In a meta-analysis of 11 intervention studies, Wewege et al.,observed that aerobic exercise, particularly of high intensity, significantly improved WC, DBP, FPG, TG, and HDL-C in individuals affected by MS but without diabetes. In contrast, resistance training did not determine significant effects [31]. A similar result was reported by Ostman et al., who in a systematic review of 16 intervention studies, found a significant improvement of anthropometric (BMI and WC) and metabolic (FPG, TG, LDL, and HDL-C) indices in adult patients affected by MS after exercise programs lasting 12 weeks or more, without differences between aerobic and combined training. The authors also evaluated the effect of the different intensity of exercise, but except for FPG, reduced only with moderate or high intensity aerobic exercise, no differences were observed regarding the other considered variables [28].

Concerning the individual components of dysmetabolism, our results are in contrast with other studies, as we did not observe any variation between the basal and final evaluation.

In particular, WC, the marker of visceral adiposity and central component of dysmetabolism, showed a significant reduction in the aforementioned studies, while in our study remained unchanged in both groups. Given the established role of visceral fat in the pathogenesis of the alterations of glucose and lipid metabolism [54], the lack of a reduction in WC measurement could be at the basis of a lack of responses in dysmetabolic people. As regards blood pressure, the medical sport evaluation showed that all people affected by dysmetabolism, and over 60% of people without dysmetabolism before the trial, also presented a mild hypertension which was clinically controlled with medical treatment. At the end of the trial, no changes were observed in people with hypertension, and none of the people without hypertension at baseline developed it during or after the trial.

However, despite the statistical significance, the improvement of individual components of MS could be less significant from a clinical point of view, especially if long-term outcomes are considered [55].

Katzmarzyk et al., evaluated the efficacy of a 20-week aerobic exercise program, finding that 30.5% of the participant with MS at baseline, did not show it at the end of the trial [56]. This result is not different from that obtained in our experimental group, but the aforementioned study lacks a control group who did not undergo the exercise training.

Another trial recruited almost one hundred people of both sexes affected by or at risk for MS and assigned them to a 6-month supervised exercise program or usual protocol. At the end of the trial, the reduction of people with MS was similar in the two groups, but only in the exercise group, none of the participants developed MS, while 7.6% in the control group developed it [57].

The latter finding is in line with our second result. Indeed, in our study, the 3-month exercise program, seemed to be effective in the prevention of dysmetabolism, since, in the experimental group, none of people without dysmetabolism at baseline, developed it at the end of the trial. In contrast, in the control group, 11.4% of individuals who initially did not have dysmetabolism, developed it at the end of the trial, with a statistically significant difference in the comparison with the experimental group.

Therefore, on the basis of our result, we can hypothesize that such an exercise program could exert a protective effect against the development of MS, and the increased associated cardiovascular risk [12]. This finding could be particularly relevant in older adults who have a higher prevalence of MS [58].

The Center for Disease Control guidelines recommend for older adults: “At least 150 min a week (for example, 30 min a day, 5 days a week) of moderate intensity activity such as brisk walking. Or they need 75 min a week of vigorous-intensity activity such as hiking, jogging, or running. At least 2 days a week of activities that strengthen muscles. Activities to improve balance such as standing on one foot, about 3 days a week. If chronic conditions affect your ability to meet these recommendations, be as physically active as your abilities and conditions allow” [45].

However, these indications are the result of a reasonable consensus rather than a properly conducted trial. Furthermore, the indications become rather vague when disorders or chronic conditions arise that may compromise the ability to perform complex exercises.

Among chronic conditions, overweight and obesity show an increasing prevalence across the world, affecting people of all ages [59], and particularly in case of abdominal adiposity, they are associated with an increased CVR [60]. In older adults, abdominal obesity, along with other cardiometabolic risk factors, is associated with a greater likelihood of frailty [61], a state of compromised homeostasis which predisposes to adverse health outcomes [62]; moreover, especially in the elderly, obesity is frequently associated with sarcopenia, generating a condition defined sarcopenic obesity [63,64]

A recent meta-analysis, including both adults and older adults, revealed a high prevalence of physical inactivity and sedentary behavior among people with obesity, and a positive association between these factors; this evidence indicates the need of a particular attention and support from health professionals [65].

In particular, based on the evidence, regular exercise is recommended for treating overweight, obesity, and MS, although it is difficult to establish the most effective exercise in terms of volume and intensity [8,66,67]. In addition, in people with obesity, the assessment of energy expenditure, crucial for determining the amount of physical activity associated with health benefits, is particularly complex [68].

Furthermore, few data are available about what type of exercise may be more effective for preventing obesity and dysmetabolism in older adults with overweight; in this field data remains largely undecided [69].

In this context, the results of the present study may represent an interesting point.

The preventive action of a medium-moderate intensity exercise on dysmetabolism, joins the preventive effect found in the same trial on psychological parameters such as depressive symptoms [38], or clinical disturbances such as chronic pain [70], and overall, an impact of such a practice on the global well-being of the elderly in a psychosomatic perspective can be underlined.

In fact, it is well known that, especially in the elderly, the two parameters can influence each other [71], and that in this age range, physical exercise can stimulate the growth of the social support network [72].

Another element that influences and can be influenced by physical activity (this one-to-one relationship is even stronger in the elderly) is proper nutrition which plays a key role in the prevention and management of weight excess and related chronic diseases [73]. Regarding MS, it has been demonstrated that a healthy dietary pattern plays a preventive role [74], and among the different dietary patterns, the Mediterranean Diet (MD) is considered one of the healthiest and easy-to follow strategies [75].

In fact, MD represents a balanced nutritional model and is able to improve the low-grade inflammation and the gut microbiota alterations typical of visceral obesity [76,77], MS, and non-communicable or inflammatory diseases [41,78,79].

Moreover, it has been reported that also an inadequate nutritional intake, commonly observed in older adults, may contribute to the development of non-communicable diseases [18].

Furthermore, it has been well established that nutritional intervention, consisting of improvement of eating habits and caloric restriction, is fundamental in order to lose weight, especially in the short term, although the best results are achieved by combining diet with exercise in the context of a long-term multidimensional treatment [34,80].

In our study, although the participants reported healthy eating habits, the anthropometric evaluation at baseline showed a mean BMI value corresponding to overweight, and WC values were higher than the sex-specific cut-off. Nevertheless, the 12-week exercise training was not associated with a nutritional program aimed at reducing body weight, as this was not an outcome of the trial.

Therefore, we hypothesize that the lack of improvement of the dysmetabolism present at baseline, could be attributed to the need to associate the exercise intervention with a dietary program aimed at reducing body weight and visceral fat, and in turn metabolic alterations. This could be considered a limitation of this study.

Another limitation, and another reason which may have contributed to the not-significant result in people affected by dysmetabolism, is that the duration of the exercise intervention was probably insufficient to reverse dysmetabolism that was already existing, especially in the case of slight metabolic alterations [81]. In fact, physical exercise parameters, such as type, intensity, frequency, and duration may differently affect the physiological responses [82,83].

On the other hand, the effectiveness of the exercise program in preventing the new onset of dysmetabolism could be ascribed to the benefits of physical activity *per se*, independent of weight loss. In this regard, it has been reported that regular exercise acts as a beneficial modulator of the low-grade inflammatory state linked to visceral adiposity and metabolic alterations [53], and of the gut microbiota composition [84] even in older adults [85].

## 5. Conclusions

This study highlights that an exercise program that is of moderate level and easily accessible in daily practice, even for older adults with a mild chronic disease such as overweight or class I obesity, prediabetes, but also controlled diabetes, dyslipidemia, or hypertension, can prevent the new onset of dysmetabolism. As for people with overweight and an already present dysmetabolism, further studies are needed that focus on the effects of a combined nutritional and exercise intervention that is more prolonged over time.

## Figures and Tables

**Table 1:** Characteristics of the two groups at baseline, before the trial (T0).

Variables	Experimental Group	Control Group	Statistics
N of individuals	**52**	**53**	χ^2^ = 0.01, *p* = 0.99
Males (%) Females (%)	23 (44%) 29 (56%)	19 (36%) 34 (64%)	χ^2^ = 0.77, *p* = 0.381
Age (years)	71.8 ± 4.7	72.7 ± 4.7	F(df1;103) = 0.76, *p* = 0.385
Years of Education	14.1± 4.6	12.7 ± 4.9	F(df1;103) = 2.27, *p* = 0.124
BMI (kg/m^2^)	26.10 ± 3.58	26.72 ± 3.59	F(df1;103) = 0785, *p* = 0.378
WC (cm) whole sample	91.98 ± 10.92	91.29 ± 13.23	*p* = 0.85
WC (cm) Males	98.05 ± 8.21	99.00 ± 7.62	*p =* 0.70
WC (cm) Females	88.17 ± 11.68	87.15 ± 13.61	*p =* 0.75
FPG (mg/dL)	89.39 ± 22.85	90.13 ± 18.03	F (df 1;103) = 0.034, *p* = 0.854
HDL cholesterol(mg/dL)	61.09 ± 13.26	60.60 ± 16.62	F (df 1;103) = 0.028, *p* = 0.868

Nominal data (sex): absolute number (%); Numerical data (age, years of education, BMI, WC, FPG, HDL-Chol): Mean ± SD. Statistics: χ^2^ test for comparison of N individuals between the two groups or between males and females; one-way ANOVA for comparison of mean values of numerical data; *p*-value < 0.05 was considered statistically significant.

**Table 2:** Effectiveness of exercise in the improvement (A) and prevention (B) of dysmetabolism after the trial (T1).

	Experimental Group (*n* 52)	Control Group (*n* 53)	Statistics
A. People with dysmetabolism before the trial (T0) A. People with dysmetabolism after the trial (T1)	15 (28.8%) 8 (15.3%)	9 (16.9%) 9 (16.9%)	* χ^2^ = 1.907 *p* =0.167 OR = 1.93 CI95% (0.76–4.93)
B. People with dysmetabolism at T1 among people without dysmetabolism at T0 (Experimental group N = 37; Control group N = 44)	0/37 (0%)	5/44 (11.4%)	χ^2^ = 4.599 *p* = 0.032 OR = INF. CL95% Not Cal

People with dysmetabolism at T0 and T1 (A), or who developed dysmetabolism at T1 (B): absolute number and percentage. Statistics: * Analysis of Variance for nominal data of Siegel and Castellan; χ^2^ test: risk of dysmetabolism at T1 among people without dysmetabolism at T0; *p* < 0.01 was considered statistically significant.

## Data Availability

The datasets are available only after requests for access directed to project leader Mauro Giovanni Carta as guarantor, according to the agreement shared with the participants and partners, and as stated in the presentation for authorization to the ethics committee.
